# 

**Published:** 1883-03

**Authors:** 


### CONTENTS:

PAGE.

ORIGINAL COMMUNICATIONS:

Antiseptic Surgery. By W. S. Tremaine, M. D., U. S. A. . . . . . . 337
On the Use of the Electro-Magnet in Removing Particles of Iron from the Eye. By Lucien Howe, M. D. . . . . . 345
Decision of the Supreme Court of the State of New York on the Question of the Validity of the Charter of the College of Physicians and Surgeons. . . . . 349
Bacillus Tuberculosis. By Mary B. Moody, M. D. . . . . . . . . . 352

SOCIETY REPORTS:

Buffalo Medical and Surgical Association, . 366
Buffalo Medical Library Association, . . 368
Constitution of the Buffalo Medical Library Association, . . . . 370
By-Laws Buffalo Medical Library Association, . . . . . . . 371

PAGE.

EDITORIAL:

The State Board of Medical Examiners, . 373
The University of Buffalo—Meeting of the Council, . . . . . . . . 376
The Alumni Association of the Buffalo Medical College, . . . . . 380
The Medical Commencement, . . 382
The Buffalo Provident Dispensary and Hospital Association, . . . . . . 383
Editorial Notes, . . . . . . . 384

REVIEWS:

A Guide to the Practical Examination of Urine for the Use of Physicians and Students, . . . . . . . 384
Pocket Therapeutics and Dose Book, . . 384

